# Metagenomics enables parallel detection of 176 clinically relevant targets from faecal samples

**DOI:** 10.3389/fcimb.2026.1759322

**Published:** 2026-02-23

**Authors:** Donovan H. Parks, Rhys J. P. Newell, Andrew N. Ginn, Kate L. Bowerman, Areej Alsheikh-Hussain, Liang Fang, Sarah Shah, Samantha MacDonald, Tristan Wimpenny, Peter Evans, Nadia E. Arias Guzman, Alena L. Pribyl, Gene W. Tyson, Philip Hugenholtz, Lutz Krause, Jim Newcombe, Paul Griffin, Michael C. Wehrhahn, Nicola Z. Angel, David L. A. Wood

**Affiliations:** 1 Microba Life Sciences Limited, Brisbane, QLD, Australia; 2 Douglass Hanly Moir Pathology, a Sonic Healthcare practice, Sydney, NSW, Australia; 3 Centre for Microbiome Research, School of Biomedical Sciences, Queensland University of Technology (QUT), Translational Research Institute, Woolloongabba, QLD, Australia; 4 Australian Centre for Ecogenomics, School of Chemistry and Molecular Biosciences, The University of Queensland, St. Lucia, QLD, Australia; 5 Mater Research, Raymond Terrace, Brisbane, QLD, Australia

**Keywords:** diagnostic test, gastrointestinal, infection, metagenomics, pathogen, antimicrobial resistance, enteropathogens, faecal

## Abstract

**Background:**

Robust identification of pathogens is essential for managing patients with symptomatic infection, yet conventional diagnostic methods focus on a subset of the most prevalent pathogens and genes. Metagenomic next-generation sequencing (mNGS) is a powerful technology that can comprehensively and simultaneously assess a broader range of pathogens and genes in a sample. This study evaluates the clinical (22 targets), analytical (19 targets), and *in silico* (176 targets) performance of a faecal mNGS assay on clinically relevant bacterial, eukaryotic, viral, virulence factor (VF) and antimicrobial resistance (AMR) genes.

**Methods:**

Diagnostic performance was evaluated relative to conventional pathology testing using 510 clinical faecal samples from patients presenting with gastrointestinal symptoms. Contrived samples were used to assess analytical performance and establish the assay’s limit of detection by adding cells to a faecal matrix. *In silico* faecal samples containing targets reflecting the limit of detection of the assay were used to evaluate performance across all 176 targets.

**Results:**

Clinical specificity was ≥96% (≥99% for all but Adenovirus F), and median pathogen sensitivity was 91%. VF and AMR gene detection was less sensitive (median 58.7%). The assay was highly reproducible in biological triplicates (27,656/27,808 calls concordant; 99.5%). Importantly, broad mNGS coverage increased diagnostic yield, with 256/510 (50.2%) samples containing one or more additional targets not reported by standard care, and 181/510 (35.5%) containing AMR genes, including carbapenemases. *In silico* benchmarking showed strong performance for all 176 targets down to analytically defined detection limits.

**Conclusions:**

The faecal mNGS assay performed competitively with existing diagnostic techniques while substantially expanding actionable detection in a single assay. These results support stool mNGS as a high-yield second-line or syndromic test for gastrointestinal infection, enabling improved recognition of rare pathogens, co-infections, and resistance determinants.

## Introduction

The reliable identification of pathogens from clinical samples is critical for guiding the management and treatment of patients with symptomatic infection. Conventional diagnostic methods used for pathogen detection include microscopy, serological assays, and molecular-based PCR assays. A primary limitation of these methods is that they focus on the most prevalent subset of pathogens and genes of clinical relevance, and often with low taxonomic resolution (e.g., genus level). As a result, patients with symptoms caused by rare or difficult-to-diagnose pathogens can remain undiagnosed or experience extended delays in receiving a diagnosis (Brett et al., 1992; Arguello et al., 2015; Kim et al., 2017; Batista et al., 2023; Nourrisson et al., 2023).

As DNA-sequencing technologies have advanced, with reduced costs, increased throughput, and improved bioinformatic tools, the use of metagenomic next-generation sequencing (mNGS) for pathogen detection has started to transition from research to clinical use (Hogan et al., 2021; Xu et al., 2023; Tan et al., 2024). mNGS can address limitations in existing infectious disease testing by providing broad coverage of pathogens, virulence factors (VF), and antimicrobial resistance (AMR) genes (Simner et al., 2018; Chiu and Miller, 2019), as demonstrated in a variety of typically sterile sample types, including blood, bronchoalveolar lavage fluid, cerebral spinal fluid, and tissue biopsies (Blauwkamp et al., 2019; Li et al., 2023; Zhang et al., 2023; Benoit et al., 2024; Fourgeaud et al., 2024). In the determination of sepsis, mNGS sequencing of blood samples more reliably identified pathogens than culture, resulting in modification of patient management and positive clinical outcomes (Blauwkamp et al., 2019; Li et al., 2023; Zhang et al., 2023). Fewer studies have used mNGS for pathogen detection in stool specimens, but preliminary studies also indicate high sensitivity and specificity are possible (Nakamura et al., 2008; Joensen et al., 2017; Peterson et al., 2022; Royer et al., 2024).

This work builds on a previous study that evaluated the diagnostic performance of a faecal mNGS assay on 11 common pathogens (Angel et al., 2024) by assessing a much broader panel of 176 targets, including pathogens (35 bacterial, 10 protozoan, two fungal, five microsporidian, 19 invertebrate, and 11 viral) and genes (45 AMR, 22 VF, and 27 host-associated AMR or VF genes). These have been chosen to cover existing highly prevalent (conventional) gastrointestinal targets and rare targets that are not well covered with existing assays. These targets were systematically validated using clinical, contrived, and *in silico* samples, subject to material availability. In total, we assessed diagnostic performance for 16 pathogens and 6 VF targets across a set of 510 clinical faecal samples, with 158 samples taken in triplicate to assess assay reproducibility. In addition, the analytical performance of the mNGS assay was assessed for three bacterial pathogens, 1 virus, and 15 AMR or VF genes by adding axenic pathogen cultures into faecal samples at concentrations spanning five orders of magnitude. Finally, performance of all 176 targets comprising the mNGS assay (**Supplementary Table S1**), the majority of which lack a validated commercially available diagnostic test, was evaluated using *in silico* faecal samples.

## Methods

### Collection of clinical samples

**Figure 1:**
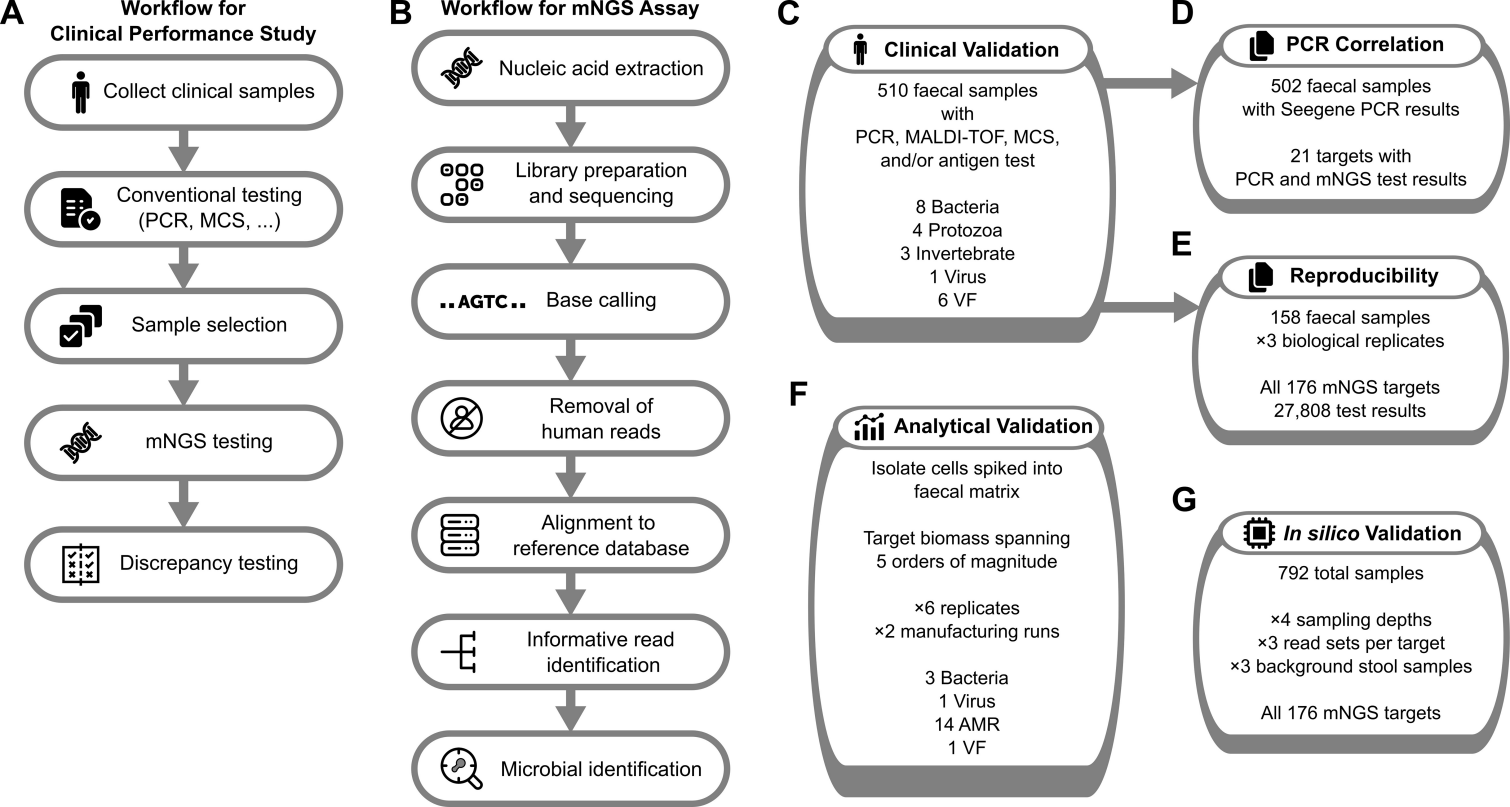
Overview of study to assess mNGS performance. Workflow for diagnostic performance study **(A)** and mNGS assay **(B)**, and the tests performed to validate the diagnostic performance **(C)**, relationship with PCR Ct values **(D)**, reproducibility **(E)**, analytical performance **(F)**, and *in silico* performance **(G)** of the mNGS assay.

Stool samples submitted to Douglass Hanly Moir Pathology (DHM) in Australia for conventional pathology testing were selected retrospectively based on positive identification of one or more targets in the mNGS assay (**Figure 1A**). These samples were from Australians with gastrointestinal symptoms referred by a treating doctor for standard infectious disease testing. Primary samples included archived material frozen at −80 °C for between 1 and 6 months, reference material, and samples undergoing contemporaneous testing. A secondary aliquot from each primary sample was taken with a Copan flocked swab (50 to 200 mg) and sent to Microba Laboratories (ISO15189 accredited) for mNGS assay testing (**Figure 1B**). DHM provided Microba with samples for 510 clinical specimens with results from one or more conventional diagnostic tests: PCR, MALDI-TOF, MCS (microscopy, culture, and sensitivity), and/or antigen test (**Figure 1C**; **Supplementary Table S2**). Additional testing was performed by Microba using Seegene PCR assays (**Supplementary Table S2**) to (i) supplement samples otherwise lacking conventional test results for study targets, (ii) evaluate the relationship between PCR cycle threshold (C_t_) values and mNGS informative reads (**Figure 1D**), and (iii) resolve discrepant results. Biological replicates were also taken from 158 samples to assess mNGS assay reproducibility (**Figure 1E**).

### Contrived samples for analytical performance

Contrived samples used to determine analytical performance were created from whole organism isolates using a homologous faecal matrix pre-screened with mNGS and PCR assays to confirm the absence of target organisms (**Figure 1F**). Analytical targets were selected to span as many different target types (bacteria, virus, VF, and AMR) as possible, accommodating the availability of reference material. The base biomass of the matrix was 4.19 to 8.67 × 10^10^ equivalent organisms/g (equ. orgs/g; avg. = 6.23 × 10^10^) assuming an average genome size of 2.8 to 5.8 Mb (average = 3.9) for stool microorganisms (Nayfach and Pollard, 2015). This aligns with the expected range for faecal material (Sender et al., 2016; Fernández-Pato et al., 2024). Isolate cells were quantified using in-house methods, which included evaluation of cells per gram of faeces with the Zymobiomics Femto Bacterial and Fungal DNA Quantification Kit (Zymo Research, CA, USA) and confirmation by microscopy-based cell counting. Calculated equivalent organisms were added to the matrix with serial dilutions covering concentrations from 10^8^ to 10^4^ orgs/g. Each set of contrived samples was processed in replicates of 6, created by different technicians using varied reagent lots on different processing runs. Two independent manufacturing runs were conducted for selected targets to verify sample creation and assay performance.

### mNGS testing of clinical and contrived samples

Samples were processed at Microba Laboratories (ISO15189 accredited) using version-controlled protocols, analytical pipelines, and reference databases (**Figure 1B**) as previously described (Angel et al., 2024). Briefly, 1 ng of genomic DNA was used for library construction using the DNA Prep Kit with DNA UD Index sets A-D (Illumina, CA, USA) and sequenced on the NovaSeq 6000 (Illumina, CA, USA) in 2 × 150 bp format, generating a minimum of 16 million non-human high-quality read pairs per sample. Each processing run met minimum sequencing quality and control sample standards, including processing of sentinel negative controls for extraction and sequencing reagents and confirmation of absence of any mNGS assay targets in these controls (see Angel et al., 2024 for additional details). Sequencing reads were filtered to remove low-quality reads using Trimmomatic (Bolger et al., 2014), and reads with high-quality alignment to the GRCh38 human genome were discarded (2.8% of reads on average). Quality-controlled reads were mapped to genomes in Microba’s reference databases, and taxonomic profiling was performed using a proprietary bioinformatic pipeline utilising the Microba Community Profiler (Parks et al., 2021) and a database indicating genomic loci that are informative of a specific target. Informative loci were identified by comparing short genomic regions across reference genomes within a genus to identify loci that are nearly ubiquitous within a target species and absent from all other species in the genus. Reads mapping to informative loci were then used as evidence for the presence of a target, which allows for the robust identification of species at low relative abundance. Target AMR and VF genes were identified by mapping reads to Microba’s reference gene database. A gene target was considered present if reads (i) covered a sufficient portion of the gene and (ii) supported all single-nucleotide variants (SNVs) required for the target to be clinically relevant (e.g., SNVs required for a gene to confer antibiotic resistance). The clinical report classifies mNGS assay targets as “detected” or “not detected” based on target-specific criteria that include the number of sequencing reads assigned to informative loci within a pathogen target, or percent coverage and identified SNVs for a gene target.

### mNGS species comprising target reference groups

Conventional tests can provide less taxonomic resolution than mNGS assays. Consequently, the specific species that produces a positive conventional test result may not be resolved. Notably, 5 of the 11 bacterial targets evaluated using Seegene PCR assays specify a target genus (e.g., *Aeromonas* spp., *Campylobacter* spp.; **Supplementary Table S3**). To evaluate the mNGS assay, targets corresponding to each reference test were established using available vendor specifications and expanded based on preliminary assessment of the mNGS assay (**Supplementary Table S3**).

### Discrepancy testing of clinical specimens

The consensus of the conventional test results was compared to the mNGS assay test result, and discrepant findings were resolved by additional testing (**Supplementary Figure S1**). Discrepancy testing was performed in-house using Seegene PCR assays for which Microba Laboratories is a certified pathology service provider. Samples were processed on the Seegene CFX96™ Real-time PCR System using the following Allplex™ assays: GI-Bacteria (I) (Cat. No. G19801Y), GI-Bacteria (II) (Cat. No. G19702Y), GI-Helminth (I) (Cat. No. GI10189Z), GI-Parasite (Cat. No. G19703Y), GI-Virus (Cat. No. G19701X), and H. pylori & ClariR (Cat. No. HC10389Z). Validation outcomes were obtainable for 61 to 497 samples depending on the target (**Supplementary Table S4**).

### Comparing PCR cycle threshold to mNGS informative reads

Cycle threshold (C_t_) values obtained by Microba Laboratories with Seegene PCR assays were compared to the number of informative reads identified by the mNGS assay, that is, reads mapping to informative loci in target species or to any portion of a target gene. Targets were considered absent for C_t_ values above 40, 43, 45, or 50 as specified by the manufacturer (**Supplementary Table S3**). The average C_t_ value was used for samples with multiple PCR test results. For reference targets with multiple mNGS assay targets (**Supplementary Table S3**), the highest number of informative reads was used.

### Generation of *in silico* faecal spike-in samples

*In silico* samples were created for all pathogen and gene targets in the mNGS assay (**Figure 1G**). Read pairs were generated at four sampling depths, corresponding to a fixed number of read pairs for pathogens and a specific coverage for genes (**Supplementary Table S5**). At the standard mNGS assay depth of 16 million read pairs, pathogen targets comprised 0.0001% (ultra-low) to 0.1% (high) of read pairs, while gene targets averaged 0.0000116% (ultra-low) to 0.000116% (high) abundance. Read pairs were generated from three independent reference genomes for each target using InSilicoSeq v1.6.0 with its NovaSeq error model (Gourlé et al., 2019), with genomes not in Microba’s reference genome databases used when available to simulate true biological variability. For targets with fewer than three genomes, independent read sets were generated using the available genomes to ensure three read sets per target. *In-silico* target reads were added to three faecal samples pre-screened to confirm they contained few mNGS assay targets, with any targets present being excluded when establishing the mNGS assay performance (**Supplementary Table S6**). Targets were divided into 22 sets covering all 176 mNGS targets to manage sample volume (**Supplementary Table S7**). A total of 792 samples were required to generate mocks at four depths, with three read sets per target added to three faecal samples.

### Performance measures

Results of the mNGS assay were compared to conventional diagnostic test results for clinical samples (**Supplementary Figure S1**), or known results for contrived and *in silico* samples to establish if a sample represented a true positive (TP), true negative (TN), false positive (FP), or false negative (FN). Performance of the mNGS assay was then assessed using sensitivity [TP/(TP + FN)], specificity [TN/(TN + FP)], positive predictive value [PPV = TP/(TP + FP)], and negative predictive value [NPV = TN/(TN + FN)]. The Clopper-Pearson exact method (Clopper and Pearson, 1934) was used to determine 95% confidence intervals (CIs) for these performance measures.

## Results

### Diagnostic performance on clinical samples

Test results from the mNGS assay were compared with conventional pathology testing for eight bacterial, four protozoa, three invertebrate, one viral, and six VF targets across 510 faecal clinical samples (**Figure 1C**; **Supplementary Table S8**). The clinical samples were obtained from children (28.8% of samples <18 years; 58.5% male) and adults (71.2% of samples; 52.3% female) presenting with gastrointestinal symptoms. Conventional testing of these faecal samples was not uniform due to technical limitations and individual clinician choice of test (**Supplementary Table S4**), and the prevalence of targets in these samples varied substantially (**Supplementary Table S9**). Using conventional pathology testing, 268 (52.5%) of the 510 clinical samples tested positive for one target, 171 (33.5%) were positive for more than one target, and 71 (13.9%) were negative for all study targets.

The mNGS assay exhibited strong performance on the majority of evaluated targets. Specificity was ≥99% for all targets except Adenovirus F (serotype 40/41) with 96.8% specificity, and the assay achieved high positive predictive value (PPV: average 98.1%; median 100.0%) and negative predictive value (NPV: average 95.7%; median 99.1%) across all targets (**Table 1**). Average sensitivity across the 16 target pathogens was 80.7% (median 91.0%) and increased to 89.0% (median 95.6%) with the removal of *Helicobacter pylori* and *Entamoeba histolytica*. The sensitivity of VFs (average 56.3%; median 58.7%) was generally lower, which can be attributed to the smaller genomic regions of these targets and, consequently, the lower likelihood of obtaining DNA sequencing reads from these regions. The broad coverage of targets in the mNGS assay resulted in 256 of 510 (50.2%) samples having 1 or more additional pathogens or VFs identified, including 83 samples with *E. coli* pathotypes and a confirmed *Tropheryma whipplei* case, compared to current standard-of-care pathology (**Supplementary Table S10**). An additional 181 samples had one or more AMR genes detected, including nine samples with carbapenemase *bla*_OXA-23_ or *bla*_OXA-48_.

**Table 1:** Performance of the mNGS assay with 95% confidence intervals (CI) for the 22 evaluated targets in the clinical dataset.

Bacteria	Positive samples	Sensitivity	95% CI	Specificity	95% CI	PPV	95% CI	NPV	95% CI
*Aeromonas* spp.	45	66.7	51.0–80.0	99.6	98.4–99.9	93.8	79.2–99.2	96.8	94.7–98.2
*Campylobacter* spp.	45	91.1	78.8–97.5	99.8	98.8–100	97.6	87.4–99.9	99.1	97.8–99.8
*Edwardsiella tarda*	3	100	29.2–100	100	99.2–100	100	29.2–100	100	99.2–100
*Helicobacter pylori*	21	4.8	0.1–23.8	100	91.2–100	100	2.5–100	66.7	53.3–78.3
*Plesiomonas shigelloides*	23	100	85.2–100	100	99.2–100	100	85.2–100	100	99.2–100
*Salmonella* spp.	40	77.5	61.5–89.2	99.8	98.8–100	96.9	83.8–99.9	98.1	96.4–99.1
*Vibrio* spp.	21	76.2	52.8–91.8	99.6	98.5–99.9	88.9	65.3–98.6	99.0	97.6–99.7
*Yersinia enterocolitica*	33	90.9	75.7–98.1	99.8	98.8–100	96.8	83.3–99.9	99.4	98.1–99.9
***Average***	28.9	75.9		99.8		96.8		94.9	
***Median***	28.0	84.2		99.8		97.3		99.1	
Eukaryotes	Positive samples	Sensitivity	95% CI	Specificity	95% CI	PPV	95% CI	NPV	95% CI
*Cryptosporidium* spp. *^#^*	36	100	90.3–100.0	99.6	98.4–99.9	94.7	82.3–99.4	100	99.2–100
*Cyclospora cayetanensis^#^*	16	100	79.4–100.0	100	99.2–100	100	79.4–100	100	99.2–100
*Entamoeba histolytica^#^*	5	40.0	5.3–85.3	100	99.3–100	100	15.8–100	99.4	98.2–99.9
*Enterobius vermicularis^&^*	9	100	66.4–100.0	100	99.2–100	100	66.4–100	100	99.2–100
*Giardia intestinalis^#^*	31	83.9	66.3–94.5	100	99.2–100	100	86.8–100	98.9	97.5–99.7
*Strongyloides stercoralis^&^*	1	100	2.5–100.0	100	99.2–100	100	2.5–100	100	99.2–100
*Taenia* spp. *^&^*	1	100	2.5–100.0	100	99.2-100	100	2.5–100	100	99.2–100
***Average***	14.1	89.1		99.9		99.2		99.8	
***Median***	9.0	100		100		100		100	
Viruses	Positive samples	Sensitivity	95% CI	Specificity	95% CI	PPV	95% CI	NPV	95% CI
Adenovirus F (serotype 40/41)	69	59.4	46.9–71.1	96.8	91.9–99.1	91.1	78.8–97.5	81.1	73.8–87.0
Virulence factors	Positive samples	Sensitivity	95% CI	Specificity	95% CI	PPV	95% CI	NPV	95% CI
*C. difficile* toxin A/B	53	64.2	49.8–76.9	100	99.2–100	100	89.7–100	95.9	93.7–97.5
EAEC virulence factors	74	75.7	64.3–84.9	99.7	98.6–100	98.2	90.6–100	95.6	93.2–97.4
EIEC virulence factors	63	60.3	47.2–72.4	100	99.2–100	100	90.7–100	94.6	92.1–96.4
EPEC virulence factors	96	30.2	21.3–40.4	100	99.0–100	100	88.1–100	84.7	81.0–88.0
ETEC heat lt/st toxins	28	57.1	37.2–75.5	100	99.2–100	100	79.4–100	97.3	95.4–98.6
STEC Shiga toxin	2	50.0	1.3–98.7	100	99.2–100	100	2.5–100	99.8	98.8–100
***Average***	52.7	56.3		99.9		99.7		94.7	
***Median***	58.0	58.7		100		100		95.8	
Overall	Positive samples		Sensitivity	Specificity		PPV		NPV	
Average for 16 pathogens	24.9		80.7	99.7		97.5		96.2	
Median for 16 pathogens	22.0		91.0	100.0		100.0		99.4	
Average for all targets	32.5		74.0	99.8		98.1		95.7	
Median for all targets	29.5		76.9	100.0		100.0		99.1	

^#^protozoa; ^&^invertebrate.

Average: average value across all targets in the group. Median: median value across all targets in the group.

PCR is the most comparable conventional diagnostic method to the mNGS assay, as both are nucleic acid tests, in contrast to MCS, MALDI-TOF, and antigen-based tests, which evaluate the viability or expression of targets. Comparing mNGS assay results to PCR resulted in sensitivity increasing to 93.9% for *Salmonella* spp. (16.4% increase), 85.0% for *Vibrio* spp. (8.8% increase), and 72.7% for *Aeromonas* spp. (6% increase) with sensitivity of *Cryptosporidium* spp. decreasing to 93.5% (6.5% decrease; **Supplementary Table S11**). Specificity, PPV, and NPV all remained the same or improved when considering just PCR as a reference diagnostic test, with the exception of NPV, which decreased slightly for *Cryptosporidium* spp. (100% to 99.3%), *Entamoeba histolytica* (99.4% to 98.3%), and *Giardia intestinalis* (98.9% to 97.1%).

### Relationship between PCR cycle threshold and mNGS informative reads

**Figure 2:**
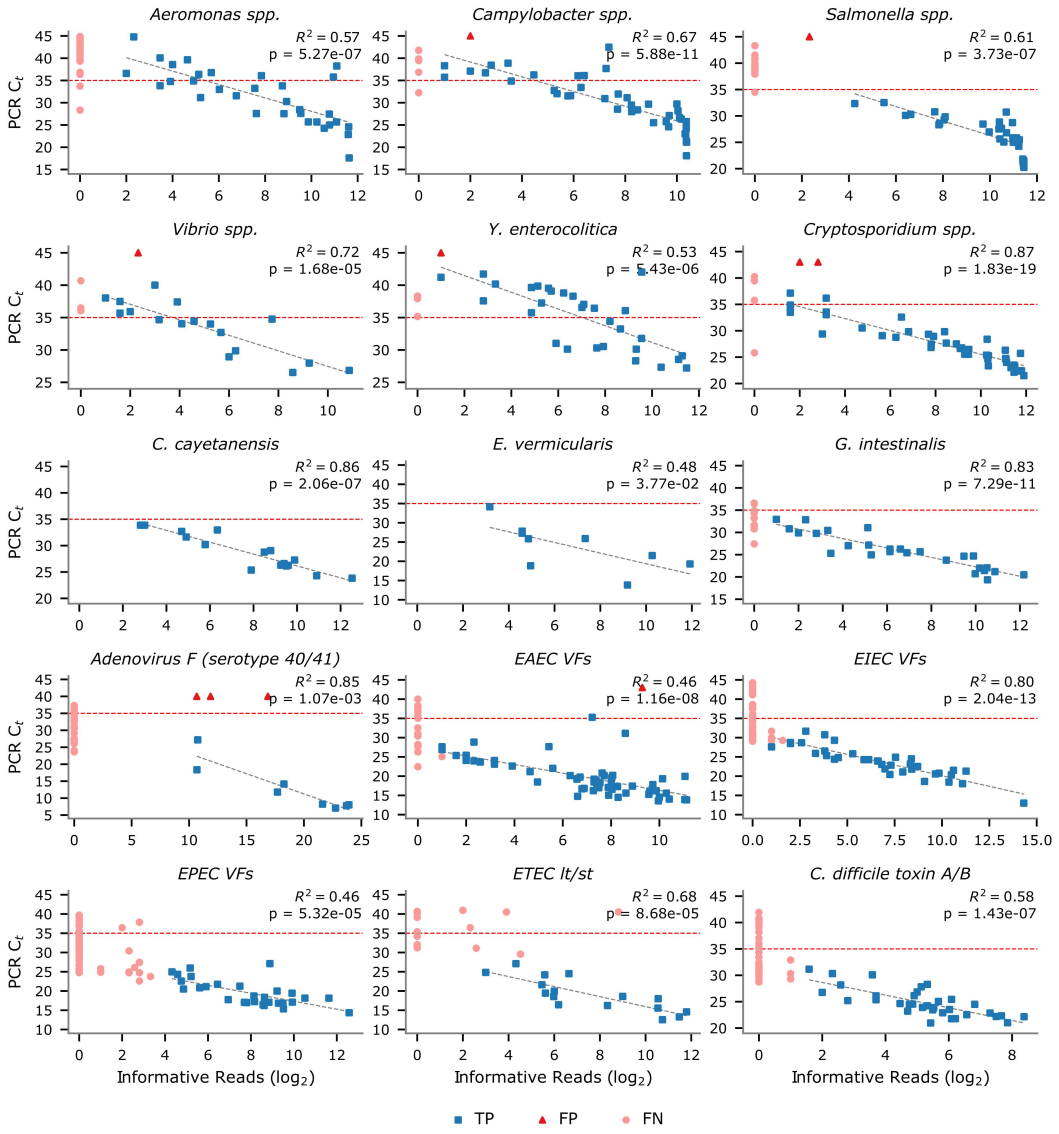
The number of informative reads (log_2_) identified by the mNGS assay used to establish the presence or absence of a target is inversely proportional to PCR cycle thresholds (C_t_ value). Each point represents a DHM sample with colour and shape indicating true positive (TP; blue square), false positive (FP; red triange), and false negative (FN; pink circle) results when comparing mNGS assay and PCR test results. The best fit line considers only TP samples and only targets with >5 TP samples are shown. The red horizontal line denotes a C_t_ value of 35.

**Figure 3:**
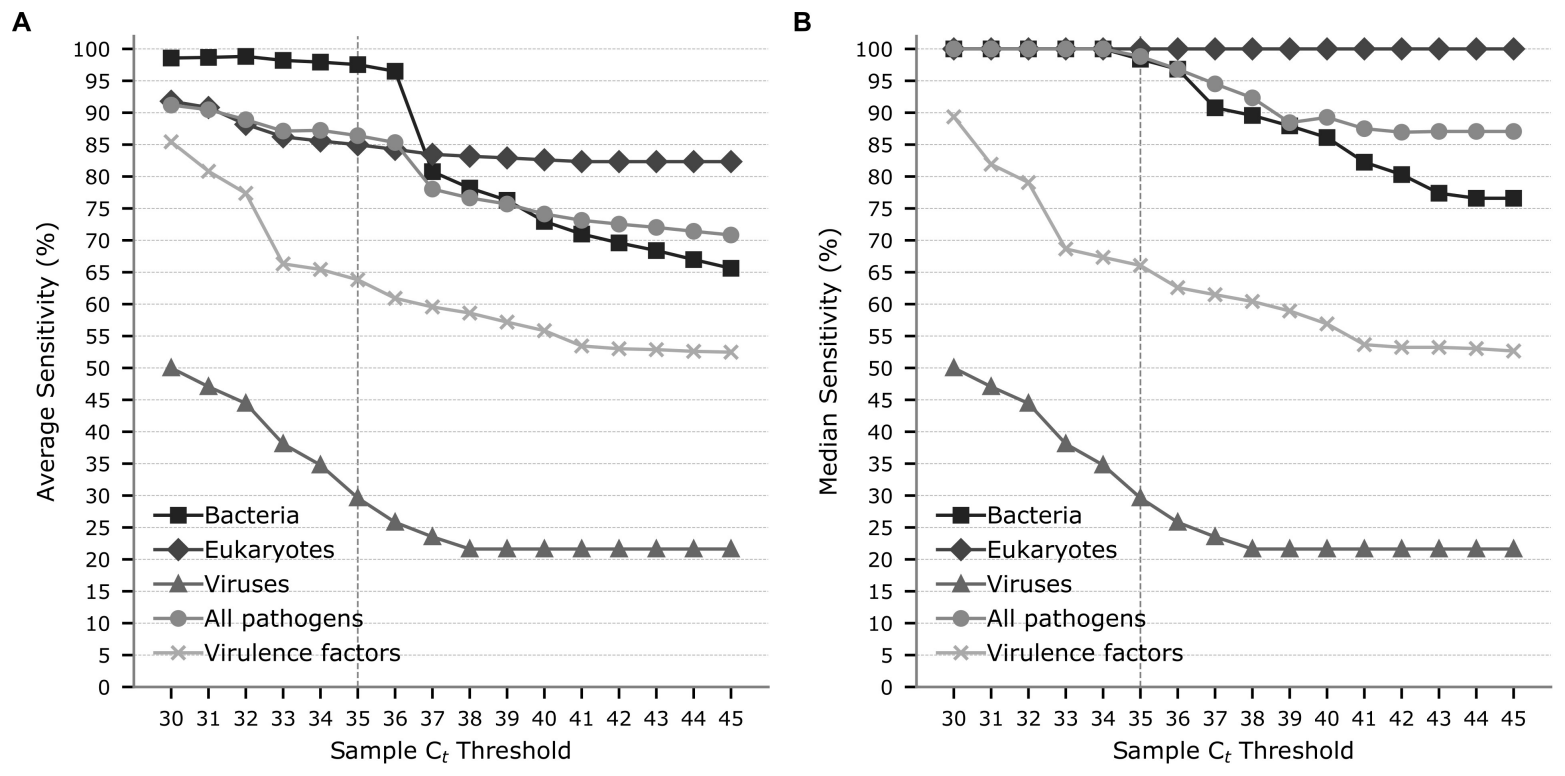
Average **(A)** and median **(B)** sensitivity of the mNGS assay when compared to test results from samples with PCR Ct values less than a specified threshold. Sensitivity is shown for targets in the groups specified in Table 1, along with results for all bacterial, eukaryotic, and viral pathogens. The grey vertical line denotes a Ct threshold of 35.

We explored the relationship between the Seegene PCR cycle threshold (C_t_) values obtained by Microba Laboratories and the number of informative sequencing reads identified by the mNGS assay (**Figure 1D**). The number of mNGS informative reads (log_2_) was found to be inversely proportional to the C_t_ value obtained by Seegene PCR assays (**Figure 2**). As expected, a substantial number of false negative (FN) results between the mNGS and PCR assays occur at high C_t_ values, as PCR test results are less reproducible at higher C_t_ values (Caraguel et al., 2011; Rhoads et al., 2021), and samples with sufficiently low pathogen load may not reach the limit of detection of the mNGS assay. Consequently, the sensitivity of the mNGS assay was found to increase steadily when compared to PCR test results on samples with a C_t_ value less than a specified threshold (**Figure 3**).

In agreement with previous studies (Boers et al., 2020), a substantial number of FN results between the mNGS and PCR assay test results were observed to occur at C_t_ values ≥35. Specifically, the sensitivity of the mNGS assay on samples with C_t_ ≥ 35 was 20.2% compared to 79.6% for samples with C_t_ < 35 (**Supplementary Table S12**). This improvement is consistent across all target groups with the average sensitivity of bacterial targets increasing from 42.3% to 97.5%, protozoan targets from 13.3% to 73.7%, Adenovirus F (serotype 40/41) from 0% to 29.6%, and VF targets from 2.0% to 63.8% (no invertebrate targets had a C_t_ ≥ 35). Targets with >20% improvements in sensitivity when considering only test results with a C_t_ < 35 as opposed to all test results include *Aeromonas* spp. (54.2% to 91.7%), *H. pylori* (6.25% to 100%), *Salmonella* spp. (68.2% to 96.8%), and the ETEC heat-labile/stable toxins (53.3% to 76.2%). Notably, *Yersinia enterocolitica* and *Campylobacter* spp. appeared to be relatively more sensitive on samples with a C_t_ ≥ 35, with 17 of 20 (85.0%) and 11 of 15 (73.3%) samples being detected, respectively; though sensitivity still improved for these targets when considering samples with C_t_ < 35 (*Y. enterocolitica* = 100%; *Campylobacter* spp. = 96.8%).

### Reproducibility of mNGS assay on clinical samples

Biological triplicates were taken for 158 clinical specimens to evaluate the reproducibility of the mNGS assay (**Figure 1E**). Reproducibility was evaluated by determining if the results of the mNGS assay were identical for a target across all three biological replicates. The assay evaluates 176 targets per specimen, resulting in a total of 27,808 (i.e., 158 specimens × 176 targets) test results of which 27,656 (99.5%) were concordant across all replicates (**Supplementary Table S13**). The majority of targets (114/176; 64.7%) were 100% or `triple/absolute’ concordant across all replicates. Of the targets with discordant results, only 2/176 (1.1%) exhibited concordance in <95% of specimens (*Campylobacter concisus*: 91.1% and *rmtD:* 89.9%).

### Analytical performance on contrived samples

Contrived samples were created for three bacterial, one viral, 14 AMR, and one VF target by adding isolate cells into the faecal matrix at equivalent organisms/g faeces decreasing from 10^8^ to 10^4^, except for Adenovirus F (serotype 40/41), where equ. orgs/g decreased from 2 × 10^10^ to 2 × 10^6^, to establish the limit of detection for a range of mNGS targets (**Figure 1F**). Contrived samples were created in replicates of 6, with select samples independently created twice, denoted by repeat (**Table 2**; see Methods). Sensitivity of target pathogens attained 100% at equ. orgs/g ≥ 10^6^ with intermittent identification of *Listeria monocytogenes* and *Pseudomonas aeruginosa* at 10^5^ equ. orgs/g. AMR and VF genes generally required 10^8^ equ. orgs/g to be robustly identified with *bla*_GES_, *bla*_lMP_, and qnrA proving challenging to identify even at this concentration. Similarly, Adenovirus F (serotype 40/41) attained 100% sensitivity at 2 × 10^8^ equ. orgs/g.

**Table 2:** Sensitivity of mNGS assay targets on contrived faecal samples at decreasing equivalent organisms/g faeces.

Equ. orgs/g					
Bacteria	10^8^	10^7^	10^6^	10^5^	10^4^
*Citrobacter freundii*	100	100	100	0	0
*Listeria monocytogenes*	100	100	100	33.3	0
*Pseudomonas aeruginosa*	100	100	100	66.7	33.3
*Pseudomonas aeruginosa* (repeat)	100	100	100	100	0
***Average***	100	100	100	50.0	8.3
Antimicrobial resistance genes	10^8^	10^7^	10^6^	10^5^	10^4^
*bla*_ACT_	100	0	0	0	0
*bla*_ACT_ (repeat)	100	0	0	0	0
*bla*_CMY_ Group 2	100	50.0	0	0	0
*bla*_DHA_	100	33.3	0	0	0
*bla*_GES_	66.7	33.3	0	0	0
*bla*_GES_ (repeat)	66.7	16.7	0	0	0
*bla*_IMP_	83.3	33.3	0	0	0
*bla*_IMP_ (repeat)	83.3	0	0	0	0
*bla*_NDM_	100	66.7	0	0	0
*bla*_NDM_ (repeat)	100	33.3	16.7	0	0
*bla*_OXA-48-like_	100	83.3	0	0	0
*bla*_VEB_	100	33.3	0	0	0
*bla*_VEB_ (repeat)	100	16.7	0	0	0
*CTX-M-G1*	100	83.3	16.7	0	0
*CTX-M-G9*	100	83.3	16.7	0	0
*qnrA*	50	0	0	0	0
*qnrB*	100	0	0	0	0
qnrB (repeat)	100	50.0	0	0	0
*rmtB*	100	16.7	0	0	0
*rmtF*	100	83.3	16.7	0	0
***Average***	86.7	35.8	3.3	0	0
Virulence factors	10^8^	10^7^	10^6^	10^5^	10^4^
*Listeriolysin O*	100	16.7	0	0	0
Viruses	2 × 10^10^	2 × 10^9^	2 × 10^8^	2 × 10^7^	2 × 10^6^
Adenovirus F (serotype 40/41)	100	100	100	0	0

Average: average value across all targets in the group. Median: median value across all targets in the group.

### Diagnostic performance on *in silico* faecal samples

*In silico* faecal samples were generated by adding *in silico* reads for all 176 mNGS targets at four read depths into three independent faecal samples in triplicate (**Figure 1G**; **Supplementary Table S5**), with ultra-low to low read depth expected to correspond to the limit of detection established using contrived samples (**Supplementary Figure S2**, **Supplementary Note S1**). The 792 *in silico* faecal samples were processed with the mNGS assay and collated to determine the performance of the assay on each target (**Supplementary Table S14**).

**Figure 4:**
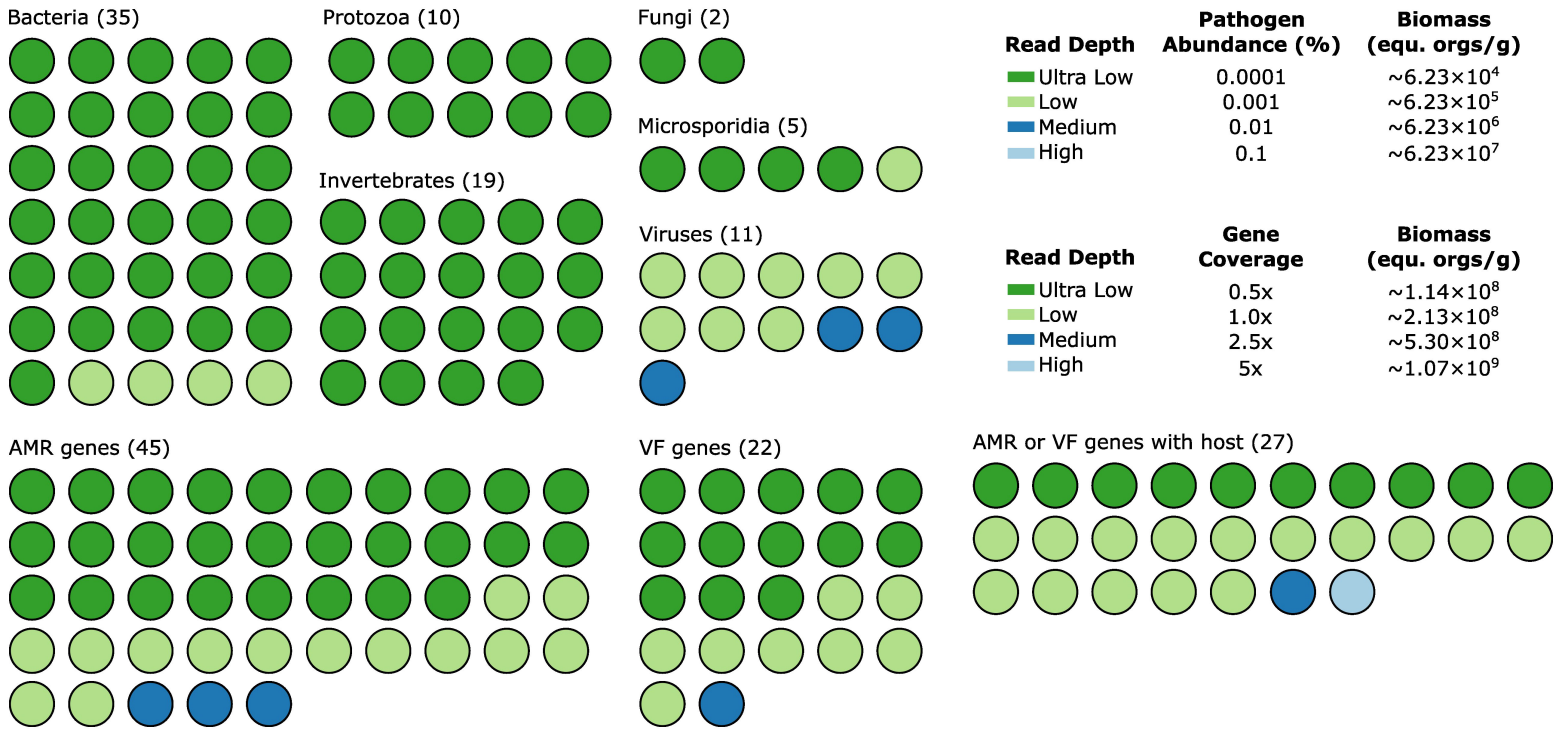
Sensitivity of the 176 mNGS assay targets on *in silico* faecal samples. Each circle represents a target coloured to indicate the lowest read depth at which 100% sensitivity was obtained. The number of targets in each category is given in parentheses.

The performance of the mNGS assay was summarised for increasing read depths for each taxonomic group of pathogens and for the AMR and VF targets (**Figure 4**). Sensitivity of pathogen targets increased with read depth with all bacterial and eukaryotic targets being identified at ultra-low depth except for *Aeromonas veronii*, *A. hydrophila*, *Salmonella bongori*, *S. enterica*, and *Anncaliia algerae* (**Table 3**; **Supplementary Table S14**). In contrast, identification of viral targets required at least low read depth (average sensitivity = 72.7%), with a few targets requiring medium read depth before they were identified and sensitivity reached 100%. Mean sensitivity was >97.8% for all gene target groups at low to high read depth (**Table 4**), with the only gene targets resulting in FN predictions above ultra-low read depth being CTX-M-G1, *bla*_KPC_, *bla*_SIM_, and EHEC O157:H7 VF genes (**Supplementary Table 14**). The mNGS assay had ≥95% mean specificity, PPV, and NPV for all pathogen and gene groups at all read depths, with these performance statistics often exceeding ≥99%. Notably, FP predictions only occurred for nine of 176 (5.1%) targets (**Supplementary Note S2**, **Supplementary Table S15**).

**Table 3:** Performance of the mNGS assay on pathogens within *in silico* faecal samples generated at increasing read depth.

	No. targets	No. samples	Average sensitivity	Average specificity	Average PPV	Average NPV
Bacteria						
Ultra Low	35	6,732	94.9	100.0	100.0	99.6
Low	35	6,732	100.0	100.0	100.0	100.0
Medium	35	6,732	100.0	99.8	97.4	100.0
High	35	6,732	100.0	99.7	97.1	100.0
Protozoa						
Ultra Low	10	1,980	100.0	100.0	100.0	100.0
Low	10	1,980	100.0	100.0	100.0	100.0
Medium	10	1,980	100.0	100.0	100.0	100.0
High	10	1,980	100.0	100.0	100.0	100.0
Fungi						
Ultra Low	2	396	100.0	100.0	100.0	100.0
Low	2	396	100.0	100.0	100.0	100.0
Medium	2	396	100.0	100.0	100.0	100.0
High	2	396	100.0	100.0	100.0	100.0
Microsporidia						
Ultra Low	5	990	80.0	100.0	100.0	99.1
Low	5	990	100.0	100.0	100.0	100.0
Medium	5	990	100.0	100.0	100.0	100.0
High	5	990	100.0	100.0	100.0	100.0
Invertebrates						
Ultra Low	19	3,762	100.0	100.0	100.0	100.0
Low	19	3,762	100.0	100.0	100.0	100.0
Medium	19	3,762	100.0	100.0	100.0	100.0
High	19	3,762	100.0	100.0	100.0	100.0
Viruses						
Ultra Low	11	2,178	0.0	100.0	n/a	95.5
Low	11	2,178	72.7	100.0	100.0	98.8
Medium	11	2,178	100.0	100.0	100.0	100.0
High	11	2,178	100.0	100.0	100.0	100.0

**Table 4:** Performance of the mNGS assay on AMR and VF genes within *in silico* faecal samples generated at increasing read depth.

	No. targets	No. samples	Average sensitivity	Average specificity	Average PPV	Average NPV
AMR						
Ultra Low	45	8,624	87.7	100.0	100.0	99.4
Low	45	8,624	97.8	100.0	100.0	99.8
Medium	45	8,624	100.0	99.9	99.1	100.0
High	45	8,624	100.0	99.8	98.0	100.0
VF						
Ultra Low	22	4,197	85.4	100.0	100.0	98.5
Low	22	4,197	99.0	100.0	100.0	99.9
Medium	22	4,197	100.0	100.0	100.0	100.0
High	22	4,197	100.0	99.1	96.1	100.0
AMR or VF with host						
Ultra Low	27	5,121	72.0	100.0	100.0	96.9
Low	27	5,121	98.4	100.0	100.0	99.7
Medium	27	5,121	98.5	100.0	100.0	99.6
High	27	5,121	98.5	99.3	96.2	99.6

Results are given separately for individual AMR and VF targets and gene targets reported with the presence of a host species.

## Discussion

This study validates the performance of an mNGS assay for detection of 176 targets in human faecal samples collected from individuals presenting with gastrointestinal symptoms (**Supplementary Table S1**). The mNGS assay exhibited acceptable diagnostic performance relative to conventional testing on the majority of the 22 targets tested, achieving >95% average and >99% median specificity, PPV, and NPV (**Table 1**). Sensitivity differed substantially between the 16 pathogens (average of 80.7%; median of 91.0%) and 6 VFs (average of 56.3%; median of 58.7%), which we attribute to the 100-fold increase in biomass required to robustly identify genes compared to pathogen targets (**Table 2**). This is evident from VF FNs being restricted to samples with high PCR C_t_ values (**Figure 2**). We predict that the low sensitivity for *H. pylori* (4.8%) detection is due to this organism typically residing in the gastric mucosa and subsequently having little residual DNA in the lower colon (Hildreth, 2008). A comparison between mNGS and PCR results demonstrated higher concordance with sensitivity for three targets increasing by >5% (**Supplementary Table S11**). This increased concordance is expected as the mNGS and PCR assays are both nucleic acid tests, in contrast to culture (often involving enrichment) and antigen-based tests, which evaluate target viability or expression. The primary measurement of the mNGS assay was shown to strongly correlate with PCR C_t_ values (**Figure 2**), and sensitivity differed substantially (20.2% to 79.6%) when comparing mNGS to PCR results with C_t_ values above or below 35, respectively. This improved sensitivity likely reflects PCR test results being less reliable at higher C_t_ values (Caraguel et al., 2011; Rhoads et al., 2021) and the mNGS assay—especially VF targets—having a lower limit of detection than PCR.

The mNGS assay was shown to be highly reproducible for the majority of targets. No target had <89.8% concordance, and targets with discordant results can be partially attributed to results near the mNGS assays’ limit of detection but may also be the result of biological heterogeneity across stool samples and indicative of the limitations of drawing conclusions based on a single swab (Hiatt et al., 1995; Branda et al., 2006). Further study is warranted to determine what portion of observed discordance reflects biological variability compared to limitations in test reproducibility.

Low-prevalence pathogens present a challenge for clinical validation of the mNGS assay, as it is not pragmatic to collect sufficient numbers of clinical samples. For example, the yearly incidence rate of Whipple’s disease (caused by *Tropheryma whipplei*), which can be fatal if left untreated, is estimated to be between 1 and 6 new cases per 10 million people (Herbay et al., 1997; Dolmans et al., 2017). Diagnostic performance of such targets can be evaluated using contrived samples, and this approach was taken to establish the limit of detection of the mNGS assay on four bacteria and 15 genes (**Table 2**). However, analytical validation is limited by the ability to source rare pathogens and difficulties in culturing organisms outside the body (Nayfach et al., 2019; Yadav et al., 2023). Broad-scale testing of large numbers of targets, regardless of their prevalence in the underlying population, can be addressed using *in silico* samples, an approach recently taken to validate a plasmid mNGS assay covering 1,250 pathogens (Blauwkamp et al., 2019). Here, we evaluated the complete set of 176 targets in the faecal mNGS assay by adding *in silico* reads from assay targets into three independent faecal samples at varying depths (**Supplementary Table 5**). Results on these *in silico* samples confirmed that all targets can be robustly identified, with targets identified at a read depth reflecting their limit of detection in the mNGS assay (**Figure 4**). A primary advantage of the mNGS assay is its broad coverage of pathogens, VF genes, and AMR genes, which in this study resulted in more than half (63.9%) of selected samples having 1 or more additional targets identified compared to current standard-of-care pathology testing, which may provide additional or more fitting clinical diagnoses for patients with persistent gastrointestinal symptoms. This assay covers DNA-based targets, and as such, commonly occurring gastrointestinal RNA viruses are not currently included. This limitation can be addressed with additional laboratory protocols, though assay cost may be prohibitive to adoption.

## Conclusions

This study demonstrates the diagnostic performance of a faecal mNGS assay on 16 pathogens and 6 VF targets, establishes that the assay provides reproducible results, and utilises *in silico* samples to assess the assay on 176 clinically relevant targets. While conventional testing such as PCR currently remains more cost-effective, mNGS assays can serve as valuable second-line tests providing diagnoses across a broad range of targets and are also capable of identification of co-infections that may include rare infections. Given the advantages of mNGS assays and the ongoing reduction in sequencing costs, we expect to see increasing adoption of these assays as part of routine clinical practice for diagnosing gastrointestinal infections in the future.

## Data Availability

The datasets presented in this study can be found in online repositories. Sequence data for clinical samples available from the National Center for Biotechnology Information under BioProject PRJNA1200893.

## Supplementary material

SUPPLEMENTARY TABLES.XLXS Supplementary tables S1, S7, S8, and S10–S15.

SUPPLEMENTARY FILE 1 Supplementary notes, tables, and figures.
