# Supplementary material for: Metagenomics enables parallel detection of 176 clinically relevant targets from faecal samples
Source: Front Cell Infect Microbiol. 2026 Feb 23;16:1759322. doi: 10.3389/fcimb.2026.1759322 (PMC12968262; doi:10.3389/fcimb.2026.1759322)
Supplement: SUPPLEMENTARY FILE 1 — Supplementary notes, tables, and figures. [file SupplementaryFile1.pdf]

Supplementary Information

Metagenomics Enables Parallel Detection of 176 Clinically Relevant Targets from Faecal Samples

Donovan H. Parks[1,*], Rhys J. P. Newell[1], Andrew N. Ginn[2], Kate L. Bowerman[1], Areej Alsheikh-Hussain[1], Liang Fang[1], Sarah Shah[1], Samantha MacDonald[1], Tristan Wimpenny[1], Peter Evans[1], Nadia Arias[1], Alena Pribyl[1], Gene W. Tyson[3], Philip Hugenholtz[4], Lutz Krause[1], Jim Newcombe[2], Paul Griffin[5], Michael C. Wehrhahn[2], Nicola Z. Angel[1], David L. A. Wood[1,*]

Supplementary Notes

Supp. Note 1. Contrived samples were used to determine the limit of detection of the mNGS assay by spiking in isolate cells at decreasing equ. orgs/g faeces into a homologous faecal matrix (see *Analytical Performance on Contrived Samples*). The faecal matrix was determined to have a biomass of 4.19 to 8.67 x 10^{10} equ. orgs/g in a faecal sample. Sensitivity reached 100% for all mNGS assay target pathogens at 10^6 equ. orgs/g (**Table 2**). This corresponds to a relative abundance of 0.00115% (100% x 10^6 / 8.67 x 10^{10}) to 0.00239% (100% x 10^6 / 4.19 x 10^{10}). For an mNGS assay operating at 16 million read pairs this corresponds to 184 to 382 read pairs from each species, assuming a genome size equal to the mean genome size of all organisms in the sample. The low depth *in silico* faecal samples were set at 160 read pairs (0.001% relative read abundance) to correspond with the expected limit of detection of a target requiring approximately 10^6 equ. orgs/g (**Supp. Table 5**). Similarly, ultra-low depth was set at 16 read pairs (0.0001% relative read abundance) to simulate targets detectable at 10^5 equ. orgs/g.

AMR and VF genes are ~1,000 bp and thus expected to have an mNGS assay limit of detection that is 3900 time lower than for a bacterial pathogen (3.9 Mb / 1000 bp). This is 3 orders of magnitude smaller which is in good agreement with the analytical results where bacterial pathogens have 100% sensitivity at 10^6 equ. orgs/g and only a subset of AMR and VF genes reach 100% sensitivity at 10^8 equ. orgs/g (i.e. indicating 10^9 equ. orgs/g or greater is required before all gene targets reach 100% sensitivity). Read pairs from AMR and VF gene targets were simulated to achieve a fixed depth of coverage between 0.5x and 2.0x as this

corresponds to an expected biomass between 1.52 x 10^7 to 2.07 x 10^9 equ. orgs/g (**Supp. Table 5**) which is around the limit of detection established for AMR and VF genes using contrived samples.

Supp. Note 2. The mNGS assay demonstrates excellent specificity and PPV across all targets as a result of few FP predictions. The limited number of FPs that occurred were investigated to establish factors resulting in incorrect predictions (126 FPs compared to 8,558 TPs and 126,635 TNs; **Supp. Table 14**). Notably, FP predictions only occurred for 9 of 176 (5.1%) targets. The only two pathogens to result in FPs were *Helicobacter cinaedi* and *H. fennelliae* and these were the results of the *in silico* samples containing the other *Helicobacter* species (i.e. *Helicobacter cinaedi* being identified in samples with *H. fennelliae*, and vice versa). There were 3 AMR, 1 VF, and 3 VF with host targets resulting in FPs. Similar, to the *Helicobacter* FPs, the FP for CTX-M-G8 is the result of the *in silico* sample contain the closely related genes CTX-M-G1 and CTX-M-G9. The cause of the other FP AMR and VF gene predictions is less clear though may be the result of interaction with DNA in the faecal background or other targets spiked into the sample (e.g. identification of the Shiga toxin in a sample that contains stx1A as a spike in and identified to contain a *Shigella/E. coli* species in the faecal background).

Supplementary Tables

Supp. Table 1. List of 176 targets included in mNGS assay and their associated category (see Excel file).

Supp. Table 2. Reference and discrepancy testing of clinical samples by DHM and Microba.

	Seegene PCR Assay								
	GI-Bacteria (I)	GI-Bacteria (II)	H. pylori	GI-Helminth	GI-Parasite	GI-Virus	MCS	MALDI-TOF	Antigen
DHM	480	0	0	0	0	162	468	468	136
Microba	476	468	51	47	470	55	0	0	0

Supp. Table 3. mNGS species or target names comprising comparison reference targets along with Seegene PCR assays and C_t thresholds.

Reference Target	*Seegene PCR Assay*	*C_t threshold*	*mNGS Assay Equivalent Targets*
Aeromonas spp.	GI-Bacteria (I)	≤45	*A. caviae, A. dhakensis, A. hydrophila, A. veronii*
Campylobacter spp.	GI-Bacteria (I)	≤45	*C. coli, C. jejuni*
Edwardsiella tarda	-	-	*E. tarda*
Helicobacter pylori	H. pylori & ClariR	≤50	*H. pylori*
Plesiomonas shigelloides	-	-	*P. shigelloides*
Salmonella spp.	GI-Bacteria (I)	≤45	*S. bongori, S. enterica*
Vibrio spp.	GI-Bacteria (I)	≤45	*V. cholerae, V. parahaemolyticus, V. vulnificus*
Yersinia enterocolitica	GI-Bacteria (I)	≤45	*Y. enterocolitica*
Cryptosporidium spp.	GI-Parasite	≤43	*C. felis, C. meleagridis, C. muris, C. ubiquitum, C. viatorum, C. hominis/parvum/cuniculus/tyzzeri complex*
Cyclospora cayetanensis	GI-Parasite	≤43	*C. cayetanensis*
Entamoeba histolytica	GI-Parasite	≤43	*E. histolytica*
Enterobius vermicularis	GI-Helminth (I)	≤45	*E. vermicularis*
Giardia lamblia	GI-Parasite	≤43	*G. intestinalis*
Strongyloides stercoralis	GI-Helminth (I)	≤45	*S. stercoralis*
Taenia spp.	GI-Helminth (I)	≤45	*T. saginata, T. solium*
C. difficile Toxin B	GI-Bacteria (I)	≤45	*C. difficile* toxin A/B
EAEC virulence factors	GI-Bacteria (II)	≤43	EAEC virulence factors
Shigella spp. / EIEC virulence factors	GI-Bacteria (I)	≤45	EIEC invasion plasmid antigen
EPEC virulence factors	GI-Bacteria (II)	≤43	EPEC virulence factors
ETEC heat labile/stable toxins	GI-Bacteria (II)	≤43	ETEC heat labile and heat stable toxins
STEC Shiga toxin	GI-Bacteria (II)	≤43	STEC Shiga toxin
Adenovirus F	GI-Virus	≤40	Adenovirus F (serotype 40/41)

Supp. Table 4. Number of samples with conventional test results for each clinical target.

Target Name	*Target Type*	*No. Samples*
Aeromonas spp.	Bacterial	497
Campylobacter spp.	Bacterial	497
Cryptosporidium spp.	Eukaryotic	490
Cyclospora cayetanensis	Eukaryotic	496
Edwardsiella tarda	Bacterial	468
Entamoeba histolytica	Eukaryotic	497
Enterobius vermicularis	Eukaryotic	468
Giardia intestinalis	Eukaryotic	497
Helicobacter pylori	Bacterial	61
Plesiomonas shigelloides	Bacterial	464
Salmonella spp.	Bacterial	497
Strongyloides stercoralis	Eukaryotic	468
Taenia spp.	Eukaryotic	468
Vibrio spp.	Bacterial	497
Yersinia enterocolitica	Bacterial	497
Adenovirus F (serotype 40/41)	Viral	193

C. difficile Toxin B	Virulence Factor	497
EAEC virulence factors	Virulence Factor	468
EIEC virulence factors	Virulence Factor	497
EPEC virulence factors	Virulence Factor	468
ETEC heat labile/stable toxins	Virulence Factor	468
STEC Shiga toxin	Virulence Factor	468

Supp. Table 5. Contrived faecal spike-in samples were generated for increasing numbers of read pairs. Pathogen targets were generated at a fixed number of read pairs whereas read pairs for gene targets were generated to achieve a fixed depth of coverage. Estimated biomass is given for a faecal sample with 6.23×10^{10} equ. orgs/g faeces assuming 3.9 Mb organisms and 1000 bp genes. Numbers in parentheses indicate the percentage of reads from a target in a standard mNGS assay sample of 16 million read pairs and considering length for the gene targets.

Depth	*Pathogen Target*	*Pathogen Biomass (equ. orgs/g)*	*Gene Target*	*Gene Biomass (equ. orgs/g)*
Ultra-low	16 (0.0001)	6.23×10^{4}	0.5 (6.25×10^{-6} to 8.75×10^{-5}; mean = 1.16×10^{-5})	1.52×10^{7} to 2.13×10^{8}
Low	160 (0.001)	6.23×10^{5}	1.0 (6.25×10^{-6} to 1.69×10^{-4}; mean = 2.11×10^{-5})	1.52×10^{7} to 4.11×10^{8}
Medium	1,600 (0.01)	6.23×10^{6}	2.5 (1.25×10^{-5} to 4.25×10^{-4}; mean = 5.75×10^{-5})	3.04×10^{7} to 1.03×10^{9}
High	16,000 (0.1)	6.23×10^{7}	5.0 (2.50×10^{-5} to 8.50×10^{-4}; mean = 1.16×10^{-4})	6.07×10^{7} to 2.07×10^{9}

Supp. Table 6. Targets identified by mNGS assay in faecal samples used to generate contrived samples with spiked-in targets.

Sample ID	*Targets Identified by mNGS Assay*
BBW5020	*Campylobacter concisus*
BBW5021	*Clostridium perfringens*
BBW5022	*blaACT, blaCMY_Group2, blaSHV, Salmonella enterica, Campylobacter concisus*

Supp. Table 7. List of mNGS assay targets in each of the 22 groups used to generate *in silico* faecal spike-in samples (see Excel file).

Supp. Table 8. Diagnostic test results for each of the 510 DHM clinical samples (see Excel file).

Supp. Table 9. Outcome of mNGS assay for each target in the DHM data set.

Bacteria	*TP*	*FP*	*TN*	*FN*	*Prevalence (%)*
Aeromonas spp.	30	2	450	15	9.05
Campylobacter spp.	41	1	451	4	9.05
Edwardsiella tarda	3	0	465	0	0.64
Helicobacter pylori	1	0	40	20	34.43
Plesiomonas shigelloides	23	0	441	0	4.96
Salmonella spp.	31	1	456	9	8.05
Vibrio spp.	16	2	474	5	4.23
Yersinia enterocolitica	30	1	463	3	6.64
**Total**	175	7	3240	56	

Eukaryotes	*TP*	*FP*	*TN*	*FN*	*Prevalence (%)*
Cryptosporidium spp.	36	2	452	0	7.35

Cyclospora cayetanensis	16	0	480	0	3.23
Entamoeba histolytica	2	0	492	3	1.01
Enterobius vermicularis	9	0	459	0	1.92
Giardia intestinalis	26	0	466	5	6.24
Strongyloides stercoralis	1	0	467	0	0.21
Taenia spp.	1	0	467	0	0.21
Total	91	2	3283	8	

Viruses	*TP*	*FP*	*TN*	*FN*	*Prevalence (%)*
Adenovirus F (serotype 40/41)	41	4	120	28	35.75

Virulence factors	*TP*	*FP*	*TN*	*FN*	*Prevalence (%)*
C. difficile toxin A/B	34	0	444	19	10.66
EAEC virulence factors	56	1	393	18	15.81
EIEC virulence factors	38	0	434	25	12.68
EPEC virulence factors	29	0	372	67	20.51
ETEC heat labile/stable toxins	16	0	440	12	5.98
STEC Shiga toxin	1	0	466	1	0.43
Total	174	1	2549	142	

All targets	*TP*	*FP*	*TN*	*FN*
Total	481	14	9192	234

Supp. Table 10. Additional targets identified by the mNGS assay compared to current standard-of-care diagnostic testing performed by DHM (see Excel file).

Supp. Table 11. Performance of the mNGS assay compared to PCR reference testing (see Excel file).

Supp. Table 12. Sensitivity of mNGS assay on samples with Seegene PCR results stratified by C_t values greater or less than 35 (see Excel file).

Supp. Table 13. Reproducibility of mNGS targets across biological replicates (see Excel file).

Supp. Table 14. Performance of each target in the mNGS assay on the *in silico* faecal spike-in samples (see Excel file).

Supp. Table 15. Exploration of FP predictions for the *in silico* faecal samples (see Excel file).

Supplementary Figures

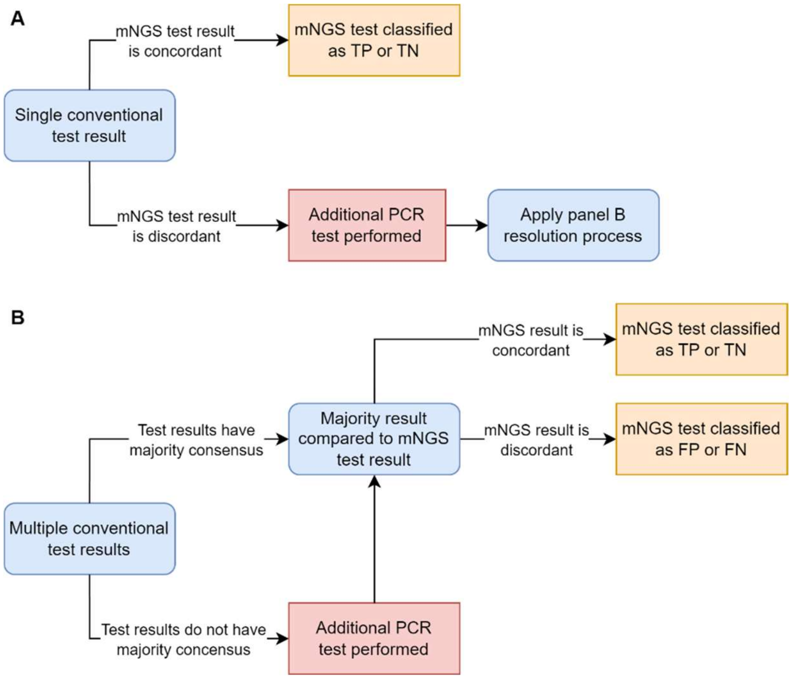

Supp. Figure 1. Decision trees for resolving discordant conventional and mNGS assay test results. Samples with a single conventional test result are either i) concordant with the mNGS assay and classified as a TP or TN, ii) discordant with the mNGS assay and subjected to an additional independent PCR test (**Supp. Figure 1A**). A majority vote (>50% consensus) was used to establish the expected result for samples provided with multiple conventional test results or where a single discordant test result prompted application of an additional independent PCR test (**B**). Samples where conventional tests had a majority result were used to evaluate the mNGS assay, whereas samples without a majority result (i.e. equal number of conventional tests indicating the absence or presence of a target) were subjected to additional PCR testing in order to establish a consensus point of truth.

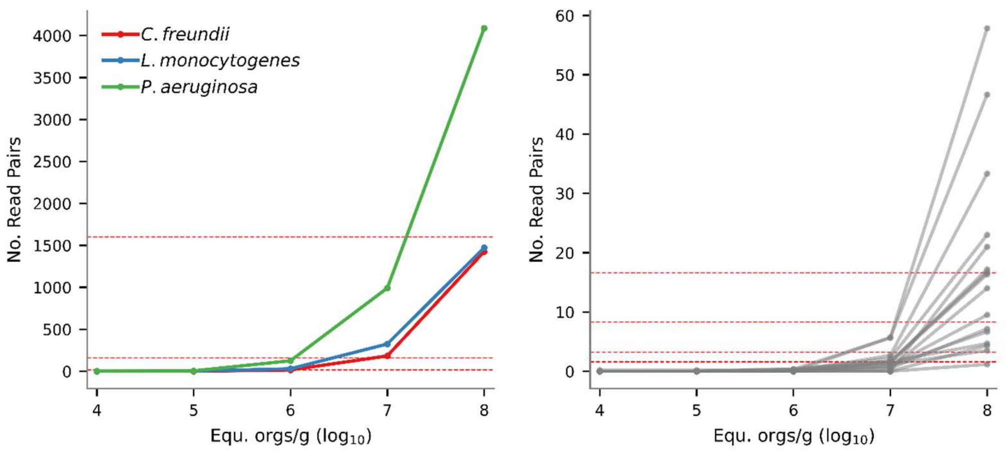

Supp. Figure 2. Number of identified reads for mNGS targets with increasing concentration in a standard faecal sample for pathogen (**A**) and gene (**B**) targets. For pathogen targets, the red dotted lines indicate the ultra-low (16 read pairs), low (160 read pairs), and medium (1,600 read pairs) read depths used for the *in silico* samples. Identification of pathogen targets required 10^5 to 10^6 equ. orgs/g faeces where the number of identified reads fall between the ultra-low and low read depths. For gene targets, the red dotted lines indicate the ultra-low (0.5x coverage; 1.6 read pairs), low (1x coverage; 3.2 read pairs), medium (2.5x coverage; 8.3 read pairs), and high (5x coverage; 16.6 read pairs) read depths used for the *in silico* samples assuming an average gene length of 1,000 bp. Robust identification of gene targets required $\geq 10^8$ equ. orgs/g faeces where the majority of targets are at or above the low *in silico* read depth.
